# Profiling and Identification of Novel Immunogenic Proteins of *Staphylococcus hyicus* ZC-4 by Immunoproteomic Assay

**DOI:** 10.1371/journal.pone.0167686

**Published:** 2016-12-08

**Authors:** Lei Wang, Zhi-wei Wu, Yan Li, Jian-guo Dong, Le-yi Zhang, Peng-shuai Liang, Yan-ling Liu, Ya-hua Zhao, Chang-xu Song

**Affiliations:** 1 College of Animal Science & National Engineering Center for Swine Breeding Industry, South China Agriculture University, Guangzhou, China; 2 Institute of Animal Health, Guangdong Academy of Agriculture Sciences, Guangzhou, China; 3 Xinyang Animal Disease Prevention and Control Engineering Research Center, Xinyang College of Agriculture and Forestry, Xinyang, China; Sun Yat-Sen University, CHINA

## Abstract

*Staphylococcus hyicus* has caused great losses in the swine industry by inducing piglet exudative epidermitis (EE), sow mastitis, metritis, and other diseases and is a threat to human health. The pathogenesis of EE, sow mastitis, and metritis involves the interaction between the host and virulent protein factors of *S*. *hyicus*, however, the proteins that interact with the host, especially the host immune system, are unclear. In the present study, immunoproteomics was used to screen the immunogenic proteins of *S*. *hyicus* strain ZC-4. The cellular and secreted proteins of *S*. *hyicus* strain ZC-4 were obtained, separated by 2D gel electrophoresis, and further analyzed by western blot with *S*. *hyicus* strain ZC-4-infected swine serum. Finally, 28 specific immunogenic proteins including 15 cellular proteins and 13 secreted proteins, 26 of which were novel immunogenic proteins from *S*. *hyicus*, were identified by matrix-assisted laser desorption ionization time-of-flight mass spectrometry. To further verify their immunogenicity, two representative proteins (acetate kinase [cellular] and enolase [secreted]) were chosen for expression, and the resultant recombinant proteins could react with *S*. *hyicus* ZC-4-infected swine serum. In mice, both acetate kinase and enolase activated the immune response by increasing G-CSF and MCP-5 expression, and acetate kinase further activated the immune response by increasing IL-12 expression. Enolase can confer better protection against *S*.*hycius* than acetate kinase in mice. For the first time to our knowledge, our results provide detailed descriptions of the cellular and secreted proteins of *S*. *hyicus* strain ZC-4. These immunogenic proteins may contribute to investigation and elucidation of the pathogenesis of *S*. *hyicus* and provide new candidates for subunit vaccines in the future.

## Introduction

*S*. *hyicus* is the major pathogen causing piglet exudative epidermitis (EE), sow mastitis, and metritis, among other diseases [1,2]. EE generally occurs as an acute infection in suckling and newly weaned piglets [3] and is characterized by greasy exudation, exfoliation, and vesicle formation [4]. We previously observed that EE led to 70%–100% mortality in non-immune farms (data not shown).

The pathogenicity of virulent bacteria is caused by the expression of numerous virulence factors [5]. Previous studies indicated that exfoliative toxin is the most important virulence factor of *S*. *hyicus* [6,7], as it can induce exfoliation or blister formation in diseased skin lesions by selectively digesting porcine desmoglein 1 directly in the porcine epidermis [8]. *Staphylococcal* protein A is another important virulence factor in *S*. *hyicus* [9]; in *S*. *aureus*, protein A binds the Fc region of immunoglobulin G [10,11] thereby inhibiting phagocytes and damaging platelets [12]. However, the pathogenic molecular mechanism of *S*. *hyicus* has not been fully clarified.

Bacterial cellular proteins [13,14] and secreted proteins [15] are necessary for cell adhesion, invasion, and pathogenicity. These proteins are all synthesized intracellularly and thereafter transported across the bacterial membrane to the bacterial cell wall or the host tissues, leading to colonization, invasion, spread, and immune responses.

Given the important role of cellular proteins and secreted proteins in bacterial pathogenicity, we employed two-dimensional gel electrophoresis (2-DE) coupled with matrix-assisted laser desorption ionization time-of-flight mass spectrometry (MALDI-TOF/TOF MS) and bioinformatics analysis to explore and identify new proteins involved in adhesion, infection, and pathogenicity of *S*. *hyicus*. Furthermore, we examined the immunogenicity of two representative proteins *in vivo* for a deeper understanding of the mechanism of *S*. *hyicus* infection.

## Materials and Methods

### Bacterial strains, culture conditions, plasmid, and animals

The highly pathogenic *S*. *hyicus* strain ZC-4 used in this study was isolated from a diseased piglet with acute EE in Guangdong province of China by our laboratory and stored at -80°C. Two types of media were used to culture ZC-4 cells at 37°C for 12h: the first was normal nutrient broth (10 g peptone, 3 g beef extract, 5 g NaCl, pH 7.4), and the second was a peptide-free medium (2.46 g MgSO_4_·7H_2_O, 17 g Na_3_PO_4_, 3 g KH_2_PO4, 0.5 g NaCl, 1 g NH_4_Cl, 4 g glucose) designed to avoid any interference by foreign proteins. *Escherichia coli* strains DH5α and BL21 and plasmid pET32a were used for cloning and prokaryotic expression. SPF mice (female and four week-old) in our study were purchased from the Experimental Animal Center of Southern Medical University, GZ, China. Twenty-five-day-old piglets were obtained from a commercial source herd negative for main pathogen (PRRSV, PRV, *Streptococcus*). After experiment finished, euthanasia was used for pigs and mice following the requirements of the animal experimental ethics.

### Preparation of swine immune serum against ZC-4

The cultured *S*. *hyicus* strain ZC-4 was centrifuged at 10,000× *g* for 3 min, washed three times, and resuspended in PBS. Twenty-five-day-old piglets were challenged with *S*. *hyicus* strain ZC-4 suspension (10^11^ CFU/mL, 3 mL/piglet) via intramuscular injection, and swine sera were collected at 15 days post-challenge and stored at -80°C for western blotting, after experiment finished, euthanasia was used for pigs.

Animal experiments were conducted in keeping with the recommendations in the Guide for the Care and Use of Laboratory Animals of the Ministry of Science and Technology of the People’s Republic of China. The present animal study was approved by the Animal Experimental Ethics Committee of the Institute of Animal Health, Guangdong Academy of Agricultural Sciences (Approval number 2012–003).

### 2-DE and western blot analysis

#### Precipitation of cellular proteins for 2-DE

Precipitation of cellular proteins from *S*. *hyicus* was performed with some modifications as described previously [16]. Briefly, *S*. *hyicus* ZC-4 was cultured to exponential-phase, centrifuged at 11,700× *g* for 20 min at 4°C, washed twice in pre-cooled PBS, and resuspended in 5 mL protein extraction buffer (40 mM Tris, 6 M urea, 2 M thiourea, 2% CHAPS (3-[(3-cholamidopropyl) dimethylammonio]-1-propanesulfonate), 50 mM DTT, 1% immobilized pH gradient [IPG] buffer, pH 3–10) with protease inhibitor mixture (2 mM EDTA, 1 mM PMSF). The suspension was incubated on ice and sonicated for 60 cycles (250 W, 2 s on, 3 s off). Cellular debris was removed by centrifugation at 15,000× *g* for 30 min at 4°C. The supernatants were cleaned using a 2-D Clean Up kit (GE Healthcare, Piscataway, NJ, USA). The concentration was determined with a 2-D Quant kit (GE Healthcare) according to the manufacturer’s instructions, and the clear supernatants were stored at -80°C for use.

#### Precipitation of secreted proteins for 2-DE

Secreted bacterial proteins were precipitated using a modified ammonium sulfate (APS) method [17]. The bacteria were cultured in nutrient broth or peptide-free medium. At exponential growth, cultures were centrifuged for 20 min at 11,700× *g* and 4°C. The supernatants were filtered through a 0.22-μm pore-size membrane filter to remove residual bacteria, APS was added to a concentration of 70% m/v, and the mixtures were incubated at 4°C overnight. After precipitation, the mixtures were centrifuged, and the pellets were resuspended in 0.01 mM PBS, dialyzed for 48 h at 4°C, and finally freeze dried. A simple cleanup and concentration step was done using the 2-D Clean Up kit and 2-D Quant kit (GE Healthcare).

#### 2-DE separation of cellular and secreted proteins

To achieve better separation, pH 3–10 IPG strips (11 cm; GE Healthcare) were used for isoelectric focusing analysis. The precipitated proteins were first treated with the 2-DE Clean-up kit and then rehydrated overnight at room temperature with rehydration solution (7 M urea, 2 M thiourea, 4% CHAPS, 50 mM DTT, 0.2% IPG buffer [pH 3–10], and 0.002% bromophenol blue). Each strip was loaded with 450 μg proteins, and 2-DE analysis was performed as described previously with modifications [18]. The samples were used to rehydrate an 11-cm IPG strip for 12 h at 20°C. The following IEF (isoelectric focusing electrophoresis)protocol was applied: 1 h at 300 V; 1 h at 600 V; 1 h at 1000 V; 1 h at 8000 V; hold at 8000 V (65,000 Vh total). After focusing was completed, IPG strips were equilibrated with 1% (w/v) DTT in equilibration base buffer containing 50 mM Tris-HCl (pH 8.8), 6 M urea, 30% glycerol, and 2% SDS for 15 min and thereafter equilibrated with 2.5% (w/v) iodeacetamide in the same buffer for 15 min. Equilibrated IPG strips were placed onto 12.5% SDS polyacrylamide gels for the second dimensional separation [16]. Two replicate 2-DE gels were used for each sample: one for Coomassie blue stain and the other for western blot analysis. Image analysis was performed with PDQuest 2-D Advance.

#### 2-DE immunoblot assays

Immunoblotting was conducted as described previously [19]. Proteins from one of the replicate 2D gels were transferred to nitrocellulose membranes (Pall, NY, USA) using transfer buffer (25 mM Tris, 192 mM glycine, 20% methanol, pH 8.3) at 250 mA for 3 h. Thereafter, the nitrocellulose membranes were washed with TBST (50 mM Tris, 150 mM NaCl, 0.05% Tween 20, pH 7.6) and blocked with 5% (w/v) bovine serum albumin (BSA) in TBST for 1 h at room temperature. Membranes were washed five times with TBST for 10 min, incubated overnight at 4°C with the anti-*S*. *hyicus* serum (1:100) in TBST containing 1% (w/v) BSA, washed another five times, and incubated with rabbit anti-swine IgG/HRP (1:8000; Invitrogen, Carlsbad, CA, USA) in TBST containing 1% (w/v) BSA for 1 h at RT. Finally the membranes were developed using an Enhanced Chemiluminescence (ECL) kit (Tiandz, Beijing, China), and images were captured by a GS800 Scanning Densitometer (Bio-Rad, CA, USA).

#### MALDI-TOF/TOF MS and bioinformatics analysis

2-DE gels and their immunoblot profiles were compared by PDQuest 2-D Advance (Bio-Rad). The immunoreactive spots were excised, and in-gel protein digestion was performed as described previously [18]. Tryptic peptides were solubilized in 0.5% trifluoroacetic acid and subjected to MALDI-TOF/TOF MS with a Bruker UltraReflexTM III MALDI-TOF/TOF mass spectrometer (Bruker Daltonics, Karlsruhe, Germany). Peptide mass fingerprints were analyzed and searched against the theoretical spectra of *S*. *hyicus*. Peptide mass fingerprinting (PMF) data were analyzed using MASCOT (Matrix Science, London, UK). MASCOT searches were used to determine the possibility of each peptide and used for the combined peptide scores. The extent of sequence coverage, number of matched peptides, and the score probability obtained from the PMF data were all used to identify proteins. Low-scoring proteins were either verified manually or rejected [20].

#### Plasmid construction, protein expression and purification

Two proteins, acetate kinase (ACK) and enolase (ENO), representing two categories of identified immunogenic proteins were chosen for prokaryotic expression. The gene fragments encoding ACK and ENO were amplified by PCR with designed primers, digested with restriction enzymes, and ligated into vector pET32a to obtain the resultant plasmids pET32a-ACK and pET32a-ENO. The constructed plasmids were transformed into *E*. *coli* strain BL21 cells, the cells were cultured at 37°C, and protein expression was induced by adding 1 mM IPTG when the OD_600_ value was 0.6–1.0. Six hours after induction, the cells were harvested, and the recombinant proteins were subjected to western Blot analysis as described above. ACK and ENO were purified with a commercial purification kit (CW Biotech, Beijing, China) according to instructions of the manufacturer, while, HIS was purified by gel electrophoresis (data not show).

#### Mouse experiments

To validate the immunogenicity of identified proteins, The BALB/c mice were injected at multiple sites intramuscularly and subcutaneously with the 200 μg purified proteins, blood samples were collected at 3 h and 24 h post injection [21,22], and cytokine concentrations were determined using the Ray Biotech mouse cytokine antibody array G2 (AAM-CYT-G2-4, Ray Biotech, Norcross, GA, USA).

Animal experiments were conducted in keeping with the recommendations in the Guide for the Care and Use of Laboratory Animals of the Ministry of Science and Technology of the People’s Republic of China. The present animal study was approved by the Animal Experimental Ethics Committee of the Institute of Animal Health, Guangdong Academy of Agricultural Sciences (Approval number 2014–010).

#### Epitope analysis of identified proteins

B-cell epitopes play a vital role in the development of peptide vaccines and in diagnosis of diseases. To map linear B-cell epitopes of the ZC-4 immunoreactive proteins screened by immunoproteomic assay [23], we used ABCPred (http://www.imtech.res.in/raghava/abcpred/) and BCPreds (http://ailab.cs.iastate.edu/bcpreds/). We employed PSORTb v.3.0.0 (http://www.psort.org/) and GposmPLoc (http://www.csbio.sjtu.edu.cn/bioinf/Gpos-multi/) to predict the subcellular localization of the proteins [24,25].

#### Immune protection test

Experiments were performed on female BALB/c mice, 26 mice were randomly divided into four groups, A: ENO (n = 5); B: ACK (n = 5); C: HIS (n = 5); D: PBS (n = 6); E: Control (n = 5). Mice were injected with 80ug of purified in complete Freund’s adjuvant, and then boosted twice, at 7 days intervals with 80ug in Freund’s incomplete adjuvant. At 7 days after the final booter injection, the blood were collected from tail vein, and then the mice were challenged with 300uL 2.8×10^9^ CFU/mL *S*.*hycius* via intramuscular and subcutaneous injection. Their percent of survival were monitored at 0, 6, 12, 20, 48, 60, and 72 h after challenge, the blood from dying mice infected by ZC-4 were collected, and incubated on blood agar plate for 20 h (HKM, GZ, China) to recovery *S*.*hycius*.

Animal experiments were conducted in keeping with the recommendations in the Guide for the Care and Use of Laboratory Animals of the Ministry of Science and Technology of the People’s Republic of China. The present animal study was approved by the Animal Experimental Ethics Committee of the South China Agricultural University (Approval number 2016–013).

#### ELISA analysis

ELISA was performed to test the antibody level, 0.25 ug purified proteins including ACK, ENO and HIS, were coated in 96 well plates (JET, GZ, China). The plates were incubates for overnight at 4°C, washed by PBST five times, and then blocked by 5% milk at 37°C for 2 h, washed by PBST five times again. After that, 100 uL of 1:10 diluted mouse anti-ACK serum, mouse anti-ENO serum or mouse anti-His serum were added to 96-well plates and incubated at 37°C for 30 min, washed by PBST five times, added 100 uL HRP-conjugated goat anti-swine IgG(H+L) as the secondary antibody, incubated at 37°C for 30 min, washed by PBST five times, added 100uL TMB (Solarbio, Beijing, China), incubated at 25°C for 10 min, added 50 uL 2M H_2_SO_4_ to stop the reaction, the absorbance was measured at 450 nm in a microplate ELISA reader (Bio-Tek, Vermont, USA).

### Statistical analysis

The significance of different groups was analyzed statistically with the Student’s t-test. The data were expressed as the mean ± standard deviation (SD), *p* values < 0.05 were considered to be significant.

## Results

### Identification of immunogenic proteins of *S*. *hyicus* ZC-4

**Fig 1:**
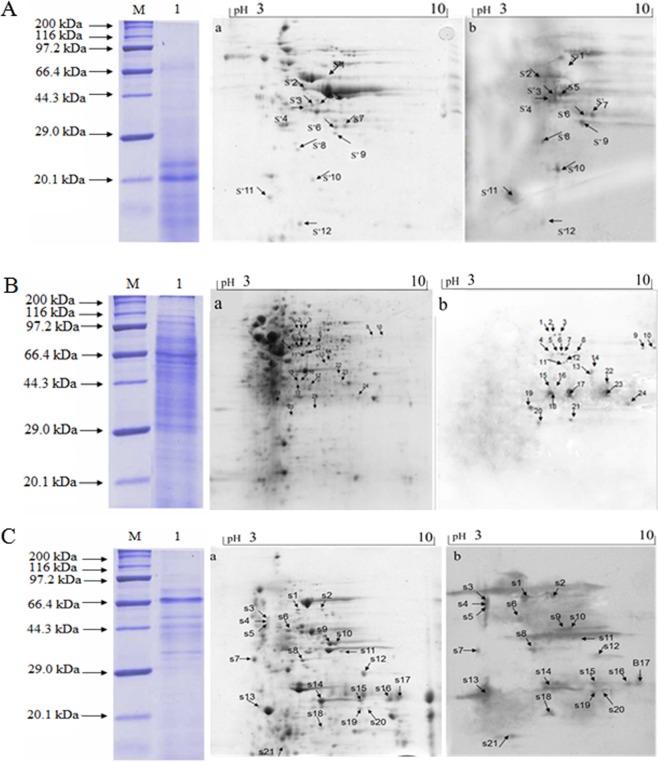
Identification of immunogenic proteins of *S*. *hyicus* ZC-4 by one or two-dimensional gel electrophoresis and western blot. Left panels: Proteins were analyzed by one-dimensional gel electrophoresis, (M) Protein marker, 20 kDa–170 kDa. (1) Proteins were analyzed by one-dimensional gel electrophoresis. Middle and Right panels: Proteins were analyzed by two-dimensional gel electrophoresis and immunoblot, all samples at 450 μg per gel were loaded onto pH 3–10 strips for electrophoresis. Middle panels: images of 2-DE gel stained with Coomassie blue (a); right panels: 2-DE gel immunostained using pig sera against ZC-4 (b). The upper to lower panels are 2-DE maps for cellular proteins (A) and secreted proteins cultured with nutrient broth (B) or with peptide-free medium (C).

To identify immunogenic proteins, samples of cellular and secreted proteins were subjected to immunoproteomics analysis, and the proteins that reacted with swine immune sera against *S*. *hyicus* ZC-4 were selected as the immunogenic proteins. We identified 24 spots from the bacterial cellular protein samples and 21 and 12 spots from bacterial secreted protein samples with normal broth and peptide-free medium, respectively, by 2-DE immunoblotting. Further analysis with MALDI-TOF/TOF MS, identified 15 cellular immunogenic proteins, seven secreted immunogenic proteins from normal broth, and nine secreted immunogenic proteins from peptide-free medium. *S*. *hyicus* lipase and phosphopyruvate hydratase (enolase) were identified from both media (Fig 1). Thus, 28 different immunogenic proteins were isolated from the *S*. *hyicus* cellular and secreted fractions; lipase and metalloprotease were identified previously [26,27], and the remaining 26 proteins were novel in this study (Tables 1 and 2).

**Table 1:** Cellular immunogenic proteins of *S*. *hyicus* ZC-4 identified by immunoproteomic assay.

Spot^1^	Protein name	Accession no.	Score^2^	MW^3^ Da	pI^4^	Loc.^5^	Pep.^6^	Cellular role	Note
2	Lysyl-tRNA synthetase	gi|323465359	76	56850	5.31	C	11	Aminoacyl-tRNA synthetase, Ligase	New
4	23S rRNA-methyltransferase (RumA)	gi|346310725	92	48312	5.56	C	11	Methyltransferase, RNA binding, Transferase	New
4	Riboflavin biosynthesis protein RibD	gi|343387788	89	41482	6.69	C	10	Riboflavin biosynthesis	New
5	Acetate kinase	gi|319892757	205	44130	5.27	C	4	Acetyl-CoA biosynthesis	New
7	Predicted protein	gi|255069905	85	25588	5.76	C	2	Glycolysis	New
8	DEAD/DEAH box helicase domain-containing protein	gi|198284110	77	117435	6.14	C	14	ATP catabolism	New
9	Phosphoenolpyruvate carboxykinase	gi|339248807	95	72186	6.51	C	9	Gluconeogenesis	New
9	Actin	gi|123298587	91	32941	5.15	C	4	Cytoskeleton organization	New
11	Methylenetetrahydrofolate dehydrogenase	gi|319892059	162	30919	5.09	C	4	Amino-acid biosynthesis; Carbon metabolism	New
12	Ornithine carbamoyltransferase	gi|242372349	102	38539	5.25	C	4	Arginine biosynthesis	New
17	Naphthoate synthase	gi|319892031	147	30574	5.39	C	7	Menaquinone biosynthesis	New
23	ATP-dependent Clp protease	gi|319891481	71	91284	5.43	C	20	Proteolysis	New

^1^: Spot number (see figures).

^2^: Mascot standard score.

^3^: Theoretical mass determined from the predicted protein sequence.

^4^: Theoretical isoelectric point determined from the predicted protein sequence.

^5^: Predicted protein localization determined using PSORTb version 3.0.0 and GposmPLoc. C, cytoplasmic; U, unknown.

^6^: Number of peptides determined using BCPreds and ABCPred.

**Table 2:** Secreted immunogenic proteins of *S*. *hyicus* ZC-4 identified by immunoproteomic assay.

Spot^1^	Protein name	Accession no.	Score^2^	MW^3^Da	pI^4^	Loc.^5^	Pep.^6^	Cellular role	Note
S1	*Staphylococcus hyicus* lipase	gi|126334	98	71237	5.89	E	14	Lipid metabolism	[26]
S2	Phosphopyruvate hydratase (Enolase)	gi|13700667	24	47145	4.55	C	7	Carbohydrate degradation, Glycolysis	New
S6	ABC transporter ATP binding protein	gi|240047779	89	123725	7.56	C	10	ATP catabolism, ATP binding	New
S7	RNA recognition motif domain containing protein	gi|156088129	92	32063	10.87	C	7	ATP hydrolysis coupled	New
S7	ATP synthase subunit C	gi|320031965	87	44367	7.74	C	6	ATP synthesis coupled, Nucleic acid binding, Oxidation-reduction process	New
S10	Conserved hypothetical protein	gi|225563124	86	94528	5.74	C	16	Unknown	New
S'1	Metalloprotease	gi|6942070	62	55828	6.13	E	12	Proteolysis, Metalloprotease	[27]
S'2	*Staphylococcus hyicus* lipase	gi|126334	98	71237	5.89	E	8	Lipid metabolism	[26]
S'4	Phosphopyruvate hydratase (Enolase)	gi|13700667	24	47145	4.55	C	7	Glycolysis, Carbohydrate degradation	New
S'8	Glucose-6-phosphate isomerase A	gi|222150838	87	49249	5.01	C	8	Glycolysis, Cellular response to oxidative stress	New
S'10	Predicted protein	gi|258572084	86	32928	6.33	C	7	Unknown	New
S'14	Isoleucine-tRNA ligase	gi|335053156	86	82573	5.21	C	18	Isoleucine-tRNA ligase, Isoleucyl-tRNA aminoacylation	New
S'15	SA2351 hypothetical protein	gi|13702514	20	57195	7.33	C	6	Carotenoid biosynthesis, Oxidoreductase activity	New
S'19	Hypothetical protein MYCGRDRAFT_41555	gi|339472387	90	122209	7.06	C	24	Lipid metabolism: Hydrolase activity, Zinc ion binding	New
S'21	SA1745 hypothetical protein	gi|15927505	37	32972	7.73	C M	2	ATP catabolism, ATP binding, ATPase activity	New

^1^: Spot number (see figures).

^2^: Mascot standard score.

^3^: Theoretical mass determined from the predicted protein sequence.

^4^: Theoretical isoelectric point determined from the predicted protein sequence.

^5^: Predicted protein localization determined using PSORTb version 3.0.0 and GposmPLoc. C, cytoplasmic; C M, cytoplasmic, membrane; E, extracellular.

^6^: Number of peptides determined using BCPreds and ABCPred.

### Functional analyses of identified immunogenic proteins

**Fig 2:**
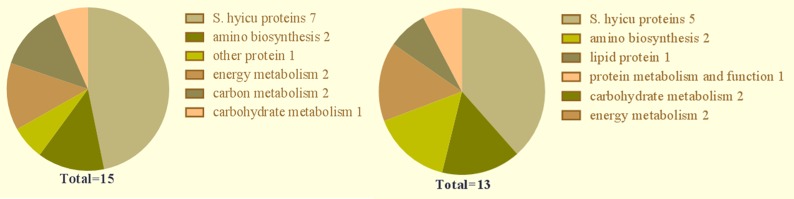
Graphical representations of immunogenic proteins categorized according to cellular function. (A) Cellular proteins. (B) Secreted proteins.

The functions of the identified immunogenic proteins of *S*. *hyicus* are summarized in Fig 2. The data demonstrated that most of the proteins were involved in amino acid transport and metabolism or energy production and conversion, and some were involved in translation, post-translational modification, protein turnover, and as chaperones.

### Immunogenicity validation of identified proteins

**Fig 3:**
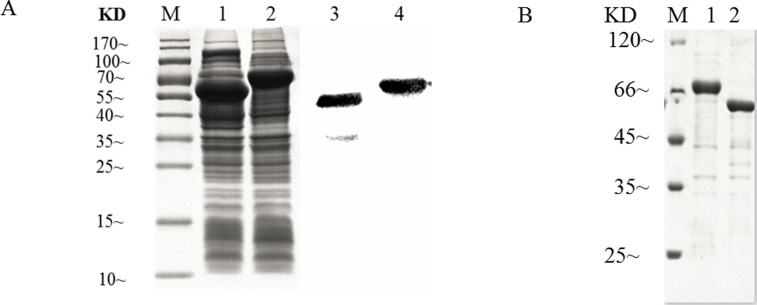
Prokaryotic expression and immunogenicity analysis of acetate kinase (ACK) and enolase (ENO). (A) Heterologous expression and western blot analysis of ACK and ENO. Lanes: M, prestained protein molecular weight marker, 10 kDa–170 kDa; 1, recombinant ACK induced by 1 mM IPTG; 2, recombinant ENO induced by 1 mM IPTG; 3, western blot analysis of recombinant ACK using ZC-4 antiserum; 4, western blot analysis of purified recombinant ENO. (B) Purification of recombinant proteins. Lanes: M, prestained protein molecular weight marker, 25 kDa–120 kDa; 1, purified recombinant ENO; 2, purified recombinant ACK.

To verify the immunogenicity of identified proteins obtained by immunoproteomic assay, ACK from cellular proteins and ENO from secreted proteins were chosen for biological function validation. The ACK and ENO proteins were expressed in *E*. *coli* and analyzed by SDS-PAGE, which confirmed that the proteins were correctly expressed with high abundance (Fig 3A). Western blot analysis of recombinant ACK and ENO demonstrated that both proteins could react with pig sera against ZC-4 (Fig 3A), suggesting that the two proteins exhibit immunogenicity. The recombinant proteins were purified on a Ni^2+^ Sepharose column for further use (Fig 3B).

### Function validation of ACK and ENO in mice

**Fig 4:**
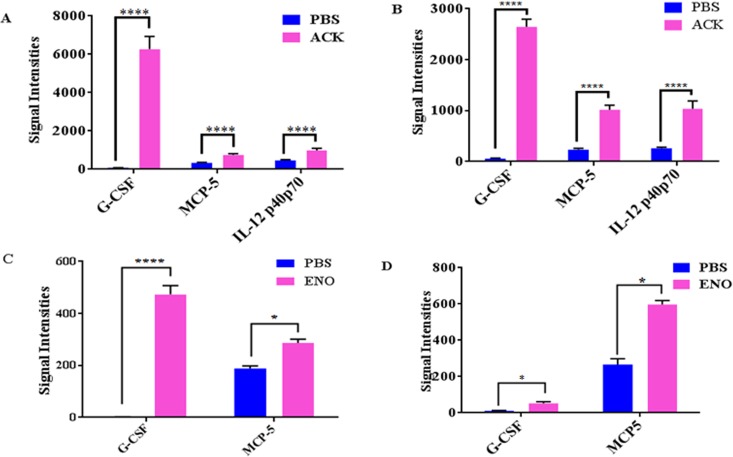
Plasma cytokine levels of BALB/c mice treated with acetate kinase (ACK) and enolase (ENO). Cytokine levels in blood samples from mice treated with recombinant ENO (A, B) or ACK (C, D). BALB/c mice were injected with the purified proteins, and blood samples were collected at 3 h and 24 h post injection for determination of cytokine levels. The significance of values were tested by GraphPad Software, “****” indicating *p*<0.001, “*” indicating *p*<0.05.

To determine the biological function of ACK and ENO, BALB/c mice were treated with purified ACK and ENO, and the levels of 32 serum cytokines including interleukin (IL)-6, IL-8, IL-12, and INF-γ were determined by protein chip at 3 h and 24 h. The levels of both G-CSF and MCP-5 increased significantly (*p* < 0.001) at 3 h and 24 h in ACK and ENO treated groups, and that the level of IL-12p40p70 increased significantly (*p* < 0.001) at 3 h and 24 h in the ACK-treated group (Fig 4).

### Epitope prediction of identified proteins

B-cell epitopes play a vital role in the antibacterial immune response and are widely used to develop peptide vaccines and diagnose diseases. We analyzed the B-cell epitopes of identified proteins, including ACK and ENO, using ABCPred and BCPreds. We identified 100 B-cell epitope antigen sequences for the cellular immunogenic proteins and 136 B-cell epitope antigen sequences for the secretory immunogenic proteins (Tables 1 and 2).

#### Immunoprotection of ENO and ACK as a subunit vaccine against *S*. *hycius* in mice

**Fig 5:**
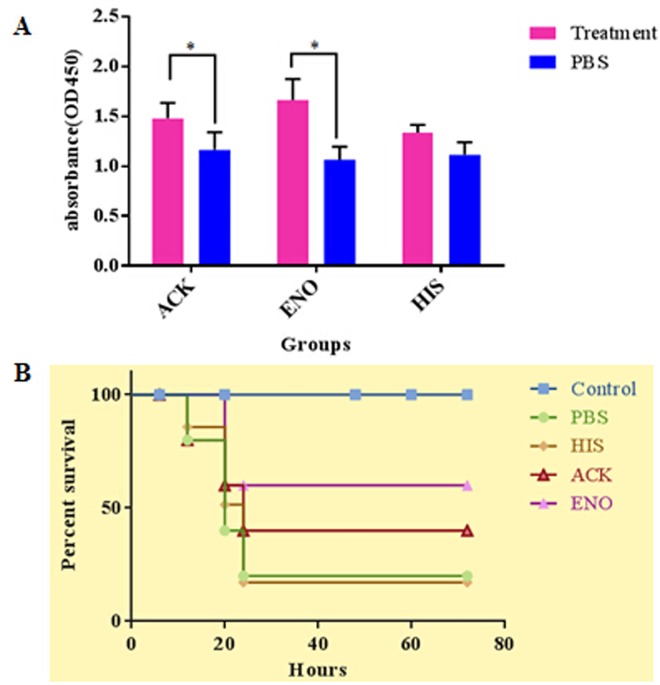
Protective immunity of ACK and ENO in mice. (A) The levels of antibody of ACK, ENO and HIS at 28 days after first immunization, measured by ELISA, the serum of PBS group was as negative control. The differences were significant between the ACK and ENO treated groups and control group, *p*<0.05 (“*”). (B) The survival percentage of ACK and ENO immuned mice. ACK, ENO, HIS and control groups had 5 mice, respectively, PBS group had 6 mice, the control group did not challenge with *S*.*hycius*. At 0, 6, 20, 48. 60. 72 post challenge hours, we observed the life-or-death situation.

Blood was collected from the tail vein of immune and control mice at 0, 7, 14, 21 and 28 days after the first immunization, and antibodies in the serum were assessed by ELISA. We wanted to screen the antibody level at 0, 7, 14, 21 and 28 days, to observe the curves of antibody, but unfortunately, the blood were too little to measure the level of antibody at these time, by ELISA(data not show), we only got the value at 28 days after first immunization (Fig 5A). The results showed that the level of antibody of treated groups were higher than that of PBS, while there was no significance between HIS treated group and PBS group, this might be because of the blood were too little, and were diluted much.

In order to evaluate the efficacy of the ENO and ACK proteins vaccine against *S*.*hycius* ZC-4 infection, the ACK and ENO immuned mice were challenged with 300uL 2.8×10^9^ CFU/mL *S*.*hycius*. The PBS and His groups mice began to die at 12 h after the challenge, after 24h, the two groups had 83.3%(5/6) and 80%(4/5) mortality. Whereas the mice in the ACK and ENO immunized groups began to die after 12 or 24 hours after the challenge, and after 24 or 36h, they had 60% (3/5) and 40% (2/5) mortality (Fig 5B), these results confirmed that the ACK and ENO immuned mice protected mice against infection by *S*. *hycius* ZC-4(Fig 5B).

## Discussion

*S*. *hyicus* is the causative agent of EE, which mainly occurs in piglets [28] with long-term clinical indicators. In particularly, because of the high temperatures and humidity in south China, there are frequent disease outbreaks that seriously damage pig farms. However, the mechanism of *S*. *hyicus* infection remains incompletely elucidated, and there is no effective vaccine for EE. Therefore, identification of the immunogenic proteins of *S*. *hyicus* and clarification of their effects on the immune response are needed for exploring the pathogenicity of pathogenic bacteria and the host antibacterial response.

Although there is no database with sufficient information about proteins in *S*. *hyicus*, we identified 28 specific immunogenic proteins from *S*. *hyicus* strain ZC-4, including 15 structure proteins and 13 secreted proteins. Of the 28 proteins, some were previously reported in other bacteria, several were subunits of multissubunitproteins, and others such as ABC transporter ATP binding protein, ENO, and ACK were novel immunogenic proteins for *S*. *hyicus*.

Among the identified bacterial cellular proteins, phosphoenolpyruvate carboxykinase, 23S rRNA (uracil-5)-methyltransferase (RumA), ornithine carbamoyl transferase, lysyl-tRNA synthetase, and ACK are appealing candidates for further study. Phosphoenolpyruvate carboxykinase can effectively induce the cell-mediated immune response by increasing CD4 T cells and cytokines such as IFN-γ, IL-12, and TNF-α, thus displaying high immunogenicity[29], and might be a promising new subunit vaccine candidate. RumA has contributed to the spread of bacterial drug resistance by interfering with initiation factor IF2 and blocking antibiotic drug binding to rRNA through the site-specific methylation of 23 rRNA by RumA [30]. Ornithine carbamoyl transferase was first discovered by Winierhoff [31] and catalyzes the carbamoylation of ornithine to form citrulline in the urea cycle. Deficiency of ornithine carbamoyl transferase leads to an X-linked aminoacidopathy characterized by hyperammonemia, neurologic abnormalities, and orotic aciduria [32]. Hughes et al. demonstrated that the major outer surface phosphoenolpyruvate carboxykinase of *Streptococcus agalactiae* could trigger the host immune response[33], our data showed that phosphoenolpyruvate carboxykinase from *S*. *hyicus* had a similar immune regulation function in pigs.

Another important protein identified in the current study was ACK. *S*. *hyicus* predominantly accumulates acetate in the culture medium, suggesting that the phosphor-transacetylase (Pta)-ACK pathway plays a crucial role in bacterial fitness. The Pta-ACK pathway in *S*. *aureus* plays an indispensable role for maintaining energetic and metabolic homeostasis during overflow metabolism [34]. Our results indicated that the recombinant ACK exhibited good immunogenicity and could react with *S*. *hyicus* antiserum. The *in vivo* mouse experiments also indicated that ACK can stimulate G-CSF, MCP-5, and IL-12 expression. IL-12 comprises covalently linked p40 and p35 subunits [35] and is an important regulator of T-helper 1 (Th1) cell responses[36]. G-CSF displays a function of enhance survival and antiapoptotic activity [37]. Mindy et al suggested that the expression of MCP-5 is involved in inflammation and the host response to pathogens [38], these proteins were upregulated in our results suggested that the *S*. *hyicus* ACK might be related to disease development. The immuneprotection test suggested that ACK can confer the week protection against *S*.*hycius* ZC-4 strain

The pathogeneses of bacteria are remarkably diverse; nevertheless, all mechanisms of bacterial incursion might be classified in three principal strategies: microbial adhesion, secretion of toxins into the extracellular milieu, and injection of virulence factors into host cells [15]. Thus, the identification of secreted proteins was of crucial importance to understanding the pathogenesis of *S*. *hyicus*.

In the present study, we identified several important secreted proteins with implications for future research. Among these, ENO has been identified several times [18,21]in other bacteria including Staphylococcus species, but this was the first report in *S*. *hyicus* to our knowledge. ENO has several auxiliary functions, e.g., as a cell-surface plasminogen receptor, as a secreted protein for a variety of pathogenic microorganisms, and as a factor in bacteria adhesion [39]. Our results showed that *S*. *hyicus* ENO can increase the cytokine levels of G-CSF and MCP-5 in mice; however, whether it is an important factor that induces IL-10 expression, as demonstrated for *Streptococcus sobrinus* ENO [21], whether or how enolase involved in S.hycius infection, all requires further study. In our study, we found that similar to enolase of *Streptococcus iniae* and *Plasmodium spp* (just enolase specific peptide sequence) [40, 41], enolase of *S*.*hycius* would confer effective protection in mice against *S*.*hycius* infection. We analyzed the homology of enolase between *S*.*hycius* and *S*. *iniae* and found that their similarity was up to 84% and higher than that betwwen *S*.*hycius* and *S*. *sobrinus* (76%). These results suggested the enolase had multifunction in different species, and in *S*.*hycius*, would be a good protective antigen. S. *hyicus* also secreted lipase, which was reported in many bacterial species as a potential contributor to colonization and persistence on the skin and is produced during bacterial infection [42–44]; Thus, lipase has been suggested as an important bacterial virulence factor and might play important role in the pathogenesis of *S*. *hyicus*. Bacterial ABC transporters are essential in cell viability, virulence, and pathogenicity. In addition to their function in transport, some bacterial ABC proteins are involved in regulation of several physiological processes. Basavanna et al. and Mei et al. provided more evidence that functioning ABC transporters were required for the full virulence of bacterial pathogens *Streptococcus pneumoniae* and *S*. *aureus* [45,46]. To the best of our knowledge, our study was the first to identify ABC transporters as secreted proteins of *S*. *hyicus*, and their function will be explored in subsequent studies. Serine/threonine protein phosphatase is involved in the DNA damage response by regulating dephosphorylation and mediating cell apoptosis[47,48]. Whether, this protease plays a role similar to that of ENO in *S*. *hyicus* invasion of host cells remains to be studied.

In addition to those discussed above, seven structural proteins and five secreted proteins reacted with antisera. So far, studies about these proteins are very limited, and the biological functions of these proteins in immune-regulation during *S*. *hyicus* infection remain unclear and require further investigation. Additional studies are necessary to elucidate the biological functions of these unknown proteins.

Interestingly, *S*. *hyicus* lipase and phosphopyruvate hydratase (ENO) were identified in both normal broth and peptide-free medium, whereas the other proteins were only secreted in one condition, indicating lipase and ENO might be essential for bacteria growth in either condition, next step, we hoped to express lipase to explore its function.

## Conclusions

In summary, we identified 28 proteins, including 15 structural and 13 secreted proteins, as specific immunogenic proteins of *S*. *hyicus* ZC-4 by immunoproteomic analysis. Two of these were identified previously, and the remaining 26 were novel for *S*. *hyicus*. Furthermore, two of these proteins, ACK and ENO from the cellular and secreted fractions, respectively, were chosen for verification by western blotting and in mouse models, which indicated that ACK and ENO had some level of immune protection against *S*.*hycius* in mice. Moreover, we analyzed the important B-cell epitopes and subcellular localizations of these proteins. This study contributes to the current understanding of the map of cellular and secreted proteins of the virulent *S*. *hyicus* ZC-4 strain and provides important information about *S*. *hyicus* proteins that can help reveal the molecular mechanisms of *S*. *hyicus* pathogenicity and develop efficient subunit vaccines.
